# Co-Occurrence of Differentiated Thyroid Cancer and Second Primary Malignancy: Correlation with Expression Profiles of Mismatch Repair Protein and Cell Cycle Regulators

**DOI:** 10.3390/cancers13215486

**Published:** 2021-10-31

**Authors:** Chih-Yi Liu, Ching-Shui Huang, Chi-Cheng Huang, Wei-Chi Ku, Hsing-Yu Shih, Chi-Jung Huang

**Affiliations:** 1 Division of Pathology, Sijhih Cathay General Hospital, New Taipei City 221, Taiwan; cyliu@cgh.org.tw; 2 School of Medicine, College of Medicine, Fu Jen Catholic University, New Taipei City 242, Taiwan; 089052@mail.fju.edu.tw; 3 Division of General Surgery, Department of Surgery, Cathay General Hospital, Taipei 106, Taiwan; cshuang@cgh.org.tw (C.-S.H.); imbighead0731@hotmail.com (H.-Y.S.); 4 School of Medicine, College of Medicine, Taipei Medical University, Taipei 110, Taiwan; 5 Comprehensive Breast Health Center, Department of Surgery, Taipei Veterans General Hospital, Taipei 1121, Taiwan; cchuang29@vghtpe.gov.tw; 6 School of Public Health, College of Public Health, National Taiwan University, Taipei 100, Taiwan; 7 Department of Medical Research, Cathay General Hospital, Taipei 106, Taiwan; 8 Department of Biochemistry, National Defense Medical Center, Taipei 114, Taiwan

**Keywords:** thyroid neoplasms, neoplasms, second primary, neoplasms, multiple primary, immunohistochemistry, DNA mismatch repair, cell cycle

## Abstract

**Simple Summary:**

Although the incidence of thyroid cancer is increasing, improvements in treatment have resulted in more patients being confirmed to have a second primary cancer. However, studies on potential biomarkers for predicting the risk of second primary malignancy are extremely limited. Therefore, our objective was to establish molecular biomarkers for the risk prediction of second primary malignancy using routinely collected formalin-fixed paraffin-embedded tissue specimens. Our results suggest that the deficient mismatch repair phenotype, the expression of pRb, and the lack of CDK4 or CDK6 are significantly associated with co-occurrence of nonthyroid malignancy. The predictive value of these immunohistochemical profiles for the co-occurrence of nonthyroid malignancy was also assessed. The combined evaluation of a four-biomarker signature model may provide the most important predictive innovation. Our study proposes the first tissue-based screening tool for risk stratification and further active surveillance in patients with thyroid cancer.

**Abstract:**

Some patients with thyroid cancer develop a second primary cancer. Defining the characteristics of patients with double primary cancers (DPCs) is crucial and needs to be followed. In this study, we examine molecular profiles in DPC. We enrolled 71 patients who received thyroid cancer surgery, 26 with single thyroid cancer (STC), and 45 with DPC. A retrograde cohort was used to develop immunohistochemical expressions of mismatch repair (MMR) proteins and cell-cycle-related markers from tissue microarrays to produce an equation for predicting the occurrence of DPC. The multivariate logistic model of 67 randomly selected patients (24 with STC and 43 with DPC) identified that the expression of deficient MMR (dMMR) (odds ratio (OR), 10.34; 95% confidence interval (CI), 2.17–49.21) and pRb (OR, 62.71; 95% CI, 4.83–814.22) were significantly associated with a higher risk of DPC. In contrast, the expression of CDK4 (OR, 0.19; 95% CI, 0.04–0.99) and CDK6 (OR, 0.03; 95% CI, 0.002–0.44) was significantly associated with a lower risk of DPC. Collectively, dMMR, pRb, CDK4, and CDK6 have a sensitivity of 88.9% (95% CI, 75.1–95.8) and a specificity of 69.2% (95% CI, 48.1–84.9) for occurrence of DPC in all 71 patients. This is the first report to demonstrate the molecular differentiation of STC and DPC. Overall, the integral molecular profile performed excellent discrimination and denoted an exponential function to predict the probability of DPC.

## 1. Introduction

Thyroid cancer is the most common type of endocrine-related malignancy. Follicular-derived or differentiated thyroid cancer (DTC) encompasses 95% of all thyroid malignancies [1,2]. In general, patients with DTC have excellent survival rates, but the prognosis highly depends on the molecular and pathological characteristics of the tumor. The molecular etiology of thyroid cancer is not fully understood. Specific genetic mutations, such as *BRAF*^V600E^, are associated with more than 70% of papillary thyroid carcinomas (PTC) [1,2]. Molecular characterization can provide valuable information to refine tumor risk stratification. Together with a prognostic role in DTC, the presence of molecular alterations can also guide targeted therapies. Therefore, a better understanding of the genetic and biologic aspects of DTC will contribute to early diagnosis and result in effective therapies and better survival outcomes.

Prolonged survival has resulted in an increased chance of nonthyroid malignancies in thyroid cancer patients, reported in several cancer registry and epidemiological studies [3,4,5,6,7,8,9,10,11,12,13,14,15,16,17,18]. The risk of developing thyroid cancer is also elevated after a variety of first primary adult cancers, but it is not clearly related to treatment [19,20,21,22]. Among Western populations, large cohort studies have shown up to 30% increase in second primary malignancies, as well as increased risks of thyroid cancer after various primary cancers [13,15,17]. In Asian countries, thyroid cancer is associated with a high rate of co-occurrence of nonthyroid malignancies [7,8]. The risk of developing second cancers after primary thyroid cancer varies from 10% to 150% depending on different types of cancer [18]. According to a population study in Taiwan, thyroid cancer is associated with a 33% risk increase for a second cancer [10]. There are sites of second cancers in the Asian population that are distinctive from those in Western populations, suggesting that genetic predisposition or environmental factors may play a role [10]. Furthermore, the co-occurrence of two primary cancers could account for 18.7% of all deaths in patients with thyroid cancer and impose a negative impact on overall survival [11]. This suggests that an increase in the incidence of double primary cancer (DPC) could be the most important late effect that occurs in patients treated for this disease.

Although several explanations for the observed elevated secondary cancer rates have been proposed, it is hypothesized that the increased risk of DPC may be related to a genetic predisposition or treatment-related complication [4,5,6,8,9,13,14,23]. Possible explanations also include active posttreatment surveillance, common environmental factors, and dietary factors [11]. In a large-scale multinational study, significantly elevated risks were observed for many specific cancers, including salivary gland, pharynx, stomach, small intestine, colorectum, bone, soft tissue sarcoma, non-melanoma of the skin, female breast, prostate, kidney, brain, adrenal gland, non-Hodgkin lymphoma, and leukemia [15]. Elevated risks of second primary thyroid cancer were also demonstrated following various specific cancers [15]. For Asian populations, there was a greater risk of developing major salivary gland, nasopharyngeal, lung, thymus, breast (female), bladder, and brain cancers, and lymphomas after diagnosis with thyroid malignancies [7,10]. The risk of a second cancer was highest within the first 5 years of thyroid cancer diagnosis and in younger patients according to a population-based study conducted in Taiwan [10]. Therefore, clinicians should maintain a high index of suspicion for the second primary after treatment for thyroid cancer.

It is important to recognize the characteristics of patients with multiple primary malignancies to detect and appropriately treat the second primary malignancy as early as possible. Although significant progress has been made in defining various molecular subtypes of thyroid cancer, the exact underlying molecular mechanism of DPC is still poorly understood. To date, very few studies have focused on the biomarker discovery aspect of DPC, and so far none has been successfully translated into clinics [24,25,26]. On the basis of our advances in understanding of carcinogenesis and knowledge of the expression of cell-cycle-related markers and the status of microsatellite instability (MSI), we can potentially develop predictive biomarkers of cancer treatment. However, comprehensive biomarker expression profiles in a large cohort of patients are difficult to obtain due to the complexity of these analyses if performed using conventional full tissue sections. In the present study, we used tissue microarray technology (TMA) combined with immunohistochemical analysis to define the importance of altered expression of these biomarkers in patients with DPC. Molecular profiles in patients with single thyroid cancer (STC) were identified and compared with the DPC group. This study explored associations between the expression of cell-cycle regulatory proteins, mismatch repair (MMR) proteins, and clinical features. The aim was then to establish molecular biomarkers for the prediction of SPC risk using routinely collected formalin-fixed paraffin-embedded tissue specimens.

## 2. Materials and Methods

### 2.1. Patients and Histopathology

This study was carried out in accordance with the Declaration of Helsinki and was approved by the Institutional Review Board of Cathay General Hospital, which granted exemption of informed consent for tissue procurement through the Cathay General Hospital Biobank after an anonymous unlinked process (IRB no.: CGH-P108136, 28 February 2020; Biobank no.: HBKEC-20200928-1).

Patients in the study were obtained from a retrospective search. The examined samples were obtained from archived tissue collections of differentiated thyroid cancer, diagnosed between 1995 and 2010. Clinical information, including age, sex, procedure to obtain the specimen, treatment modality, and follow-up information, was obtained from the biobank database. All samples examined in this study were formalin-fixed paraffin-embedded (FFPE) samples of thyroid cancer.

Patients with a second cancer in organs other than the thyroid were enrolled (double primary cancer (DPC), *n* = 45), and those without available FFPE tissue samples were excluded. To compare the characteristics between the single thyroid cancer (STC) and DPC groups, the STC group was selected from thyroid cancer patients who were not diagnosed with prior or subsequent nonthyroid cancers during the follow-up period (*n* = 26). A total of 71 patients were enrolled in this study and all their available histological slides were reviewed to confirm the diagnosis of DTC. Then, 67 patients (24 with STC, 13 papillary carcinoma and 11 follicular carcinoma; 43 with DPC, 35 papillary carcinoma and 8 follicular carcinoma), randomly selected from these 71 patients, served as cases to establish the predictive model. The median age of these 67 patients (12 men and 55 females) was 52 years (range, 27 to 82 years). The prediction of the primary model derived from 67 patients was used to check the probability of DTC of all 71 patients.

### 2.2. Tissue Microarray (TMA) Construction

The archive pathology specimens of the 71 patients underwent a pathology review to evaluate histological tumor characteristics and to select the representative area of the specimen to be included in the TMA. Briefly, a 3-mm core of the paraffin-embedded tissue was removed from the preselected region of each donor tissue block and then assembled into a high-density TMA. Subsequent immunohistochemical analysis of nine biomarkers and four MMR proteins was carried out on TMA blocks, as described below.

### 2.3. Immunohistochemistry (IHC)

Sections of 5 μm were cut from the TMA blocks then processed using the avidin–biotin–immunoperoxidase method. Immunohistochemical assays were performed on a BenchMark XT slide stainer (Ventana Medical Systems, Tucson, AZ, USA). The automated immunohistochemistry program included deparaffinization, antigen retrieval, incubation with primary antibody followed by secondary antibody, and visualization of chromogen. External tissue controls were used as positive and negative controls to check for false positive or false negative results. Negative reagent controls were performed to ensure that staining is produced by detection of the antigen by the primary antibody (Supplementary Figure S1).

For each case, monoclonal antibodies to p21 (clone DCS-60.2, 1:100; Cell Marque), cyclin D1 (clone SP4-R, ready to use; Roche Ventana), p16^INk4a^ (clone E6H4, ready to use; Roche Ventana), pRb (clone 13A10, 1:100; Leica Biosystems), CDK2 (clone E304, 1:100; Abcam), CDK4 (clone C-22, 1:100; Santa Cruz), CDK6 (clone EPR4515, 1:100; Abcam), E2F1 (clone KH95, 1:100; Santa Cruz), and Ki-67/MIB-1 (clone 30-9, ready to use; Roche Ventana) were used.

To investigate mismatch repair (MMR) protein deficiency, immunohistochemical stains for the four MMR proteins were performed on the Ventana Benchmark autostaining system. Primary antibodies for MLH1 (clone M1, ready to use; Roche Ventana), PMS2 (clone EPR3947, ready to use; Roche Ventana), MSH2 (clone G219-1129, ready to use; Roche Ventana), and MSH6 (clone 44, ready to use; Roche Ventana) were applied.

The slides were scanned on an Aperio Image Scopeslide scanner and then analyzed using Image Scope software (Aperio Technologies, Vista, CA, USA). The use of a scanner is particularly helpful for analyzing TMA slides. It provides excellent image quality while allowing for better viewing of the numerous tissue cores present in a single slide.

### 2.4. IHC Interpretation

A pathologist, blinded to clinical data, examined the sections with high power to determine the proportion of cells that express the markers. For each marker, the entire tumor core section was evaluated. The cut-off values used in this study were based on previously established cut-off values from immunohistochemical studies for the nine markers [27,28,29,30,31]. The cut-off values for tumor cell staining were defined as follows: (1) high expression of cyclin D1 if ≥50% of tumor nuclei stained; (2) high expression of p21 if ≥50% of tumor nuclei stained; (3) positive for pRb if ≥10% of tumor nuclei stained; (4) positive for p16^INk4a^ if ≥10% of tumor nuclei stained; (5) positive for CDK2 if ≥10% of tumor nuclei stained; (6) positive for CDK4 if ≥10% of tumor nuclei stained; (7) positive for CDK6 if ≥10% of tumor nuclei stained; (8) positive for E2F1 if ≥10% of tumor nuclei stained; and (9) high Ki-67 proliferative index if equal or more than 5% of tumor nuclei stained.

For the definition of the MSI screening status, loss of MMR protein expression was defined as the complete absence of nuclear staining throughout the tumor area. The lymphocytes and vascular endothelial cells served as a positive internal control. If one or more of the MMR proteins were not expressed, the result was referred to as deficient mismatch repair (dMMR).

**Figure 1:**
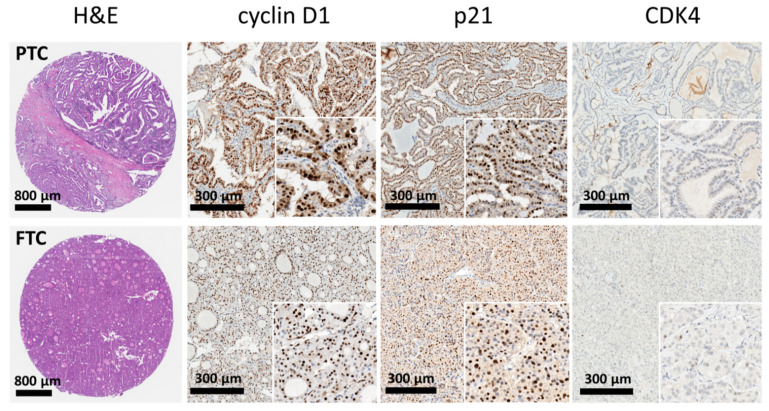
Representative images and distribution of the investigative markers. The microarray cores exhibited features of papillary carcinoma (**upper panels**) and follicular carcinoma (**lower panels**). The cores of thyroid cancer tissue showed strong expression of cyclin D1 and p21, while loss of expression of CDK4.

Representative immunophenotypes for the selected marker investigated in tumor tissue are shown in Figure 1.

### 2.5. Statistical Methods

The basic demographics of the patients between the STC and DPC groups were compared using Fisher’s exact test. To evaluate the association between biomarkers (including mismatch repair (MMR) proteins and cell-cycle regulatory proteins) and DPC status, univariate logistic regression analyses were initially performed. Moreover, those significant biomarkers in the univariate analyses were introduced into a multivariate model with backward elimination. The predicted probability of having a nonthyroid cancer derived from the multivariate model was further treated as an explanatory variable in the receiver operating characteristic (ROC) curve analysis. Univariate logistic regression analyses were stratified by tumor subtype to assess whether the relationship between each biomarker and DPC status was consistent in both subtypes. Lastly, to investigate the potential additive effect among significant biomarkers, the interaction effects among the significant biomarkers were tested. All tests were two-tailed and *p* < 0.05 was considered statistically significant. Data analyses were performed using SPSS 25 (IBM SPSS Inc., Chicago, IL, USA).

## 3. Results

### 3.1. Clinical Features of DTC Patients

To establish a prediction model, the cohort consisted of 24 patients with STC, as well as those with cancers in organs other than the thyroid (43 patients with DPC). The patient characteristics at baseline are presented in Table 1. Among the 24 SPC cases, nine patients (37.5%) were alive, while death from cancer or non-cancer-related causes was found in five patients (20.8%) during follow-up.

Of the 43 DPC cases, nine were synchronous (interval of diagnosis <6 months), while 34 were metachronous (interval of diagnosis >6 months). The second primary malignancy included tumors from the following sites: colorectum (*n* = 12, 27.9%), breast (*n* = 11, 25.6%), head and neck (*n* = 6, 14.0%), lung (*n* = 6, 14.0%), gynecologic tract (*n* = 4, 9.3%), soft tissue (*n* = 2, 4.7%), prostate (*n* = 1, 2.3%), and lymphoma (*n* = 1, 2.3%). Up to the last date of follow-up, 29 patients (67.4%) were alive, while death from cancer or noncancer-related causes was found in eight patients (18.6%).

### 3.2. Expression of Mismatch Repair (MMR) Proteins and Cell-Cycle Regulatory Proteins

**Figure 2:**
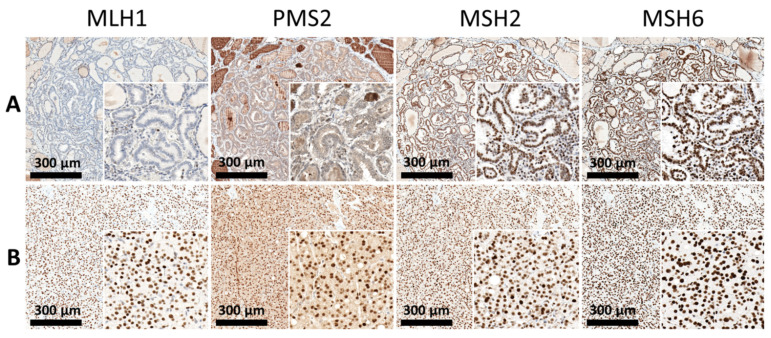
Expression pattern of MMR proteins. Panel (**A**): The tumor sample from papillary carcinoma showed dMMR status with complete loss of nuclear staining for MLH1 and PMS2. Panel (**B**): The tumor sample from follicular carcinoma exhibited an MMR-proficient status with intact nuclear staining for MMR proteins.

Immunohistochemically, dMMR was defined as complete loss of expression in one of PMS2, MLH1, MSH2, or MSH6. Of the 24 patients in the STC group, eight (33.3%) showed dMMR, with loss of expression of at least one MMR protein. The features of dMMR were identified significantly more frequently in 32 (74.4%) patients with DPC. Overall, in the abnormal IHC group, the most common deficiency identified was the simultaneous loss of expression of MLH1 and PMS2 in 23 patients, followed by isolated loss of PMS2 in 13 patients and the absence of MSH2 and MSH6 in four patients. Furthermore, all patients with DPC were further divided into groups of dMMR (32 patients) (Figure 2A) and MMR-proficient groups (intact MMR, 11 patients) (Figure 2B).

Table 2 presents the results related to the immunohistochemical expression of the markers examined. The overall positive expression rates of pRb, p16^INk4a^, CDK2, CDK4, CDK6, and E2F1 in the STC group were 25.0%, 12.5%, 12.5%, 54.2%, 33.3%, and 33.3%, respectively. A high expression of p21 (>50%) was observed in 20.8% of cases, while a high expression of cyclin D1 (>50%) was observed in 54.2%. Most STC tissues (91.7%) revealed a low Ki-67 index (<5%).

The overall positive expression rates of pRb, p16INk4a, CDK2, CDK4, CDK6, and E2F1 in the DPC group were 60.5%, 30.2%, 44.2%, 20.9%, 9.3%, and 34.9%, respectively. A high expression of p21 (>50%) was observed in 46.5% of cases, while a high expression of cyclin D1 (>50%) was observed in 74.4%. An increase in the Ki-67 index (>5%) was observed in 32.6% of patients with DPC.

Immunoreactivities for cell-cycle regulatory proteins in the DPC group revealed that cyclin D1 and p21 were found to be more expressed than in the STC group. Positive staining for pRb and CDK2 also increased in the DPC group. In contrast, the lack of expression of CDK4 or CDK6 was found to be significantly more common in the DPC group. The DPC group showed a propensity for a high Ki-67 proliferative index.

**Figure 3:**
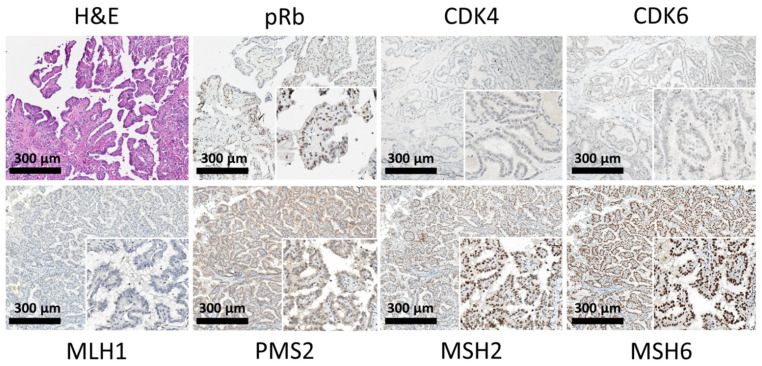
Representative immunohistochemical staining for the expression of pRb, CDK4, CDK6, and MMR proteins in the double primary cancer (DPC) group. Papillary carcinoma (left upper panel; H&E stain) showed high expression of pRb, while most cancer cells were negative for CDK4 and CDK6. Tumor tissue demonstrated deficient mismatch repair (dMMR) with loss of MLH1 and PMS2 expression.

Univariate analyses showed that the expression of the following biomarkers was correlated with the status of DPC: dMMR, cyclin D1, p21, pRb, CDK2, and Ki-67. On the other hand, univariate analyses indicated that CDK4 and CDK6 expression was correlated with STC status. The backward elimination multivariate model demonstrated that the expression of dMMR (odds ratio (OR) 10.34, 95% confidence interval (CI) 2.17–49.21) and pRb (OR 62.71, 95% CI 4.83–814.22) was significantly associated with the status of DPC. In contrast, the expression of CDK4 (OR 0.19, 95% CI 0.04–0.99) and CDK6 (OR 0.03, 95% CI 0.002–0.44) was significantly associated with the status of STC (Table 2). Representative immunohistochemical images of the investigative markers in the TMA of the DPC group are shown in Figure 3.

### 3.3. Immunoexpression Patterns in the Papillary Carcinoma (PTC) and Follicular Carcinoma (FTC) Subtypes and the Interaction between the SPC and DPC Groups

Table 3 presents the association between biomarker expression and STC or DPC status, stratified by subtypes PTC and FTC. Most of the results were less significant due to the smaller sample size in each tumor subtype group. It is noted that all interaction effects between each biomarker and tumor subtypes were not significant, indicating that the tumor subtypes did not modify the association between each biomarker and the status of STC or DPC. In other words, the relationship between the MMR/cell-cycle regulatory proteins and the status of STC or DPC was similar between the PTC and FTC subtypes.

### 3.4. Immunoexpression Patterns of DTC and the Interrelationship with Significant Biomarkers

The previous multivariate model identified four significant biomarkers: dMMR, pRb, CDK4, and CDK6 (Table 2). The interrelationship among the four important biomarkers is of interest (Table 4). The results showed that the interaction effects between each significant biomarker and the other three significant biomarkers were not significant, suggesting that the association between a significant biomarker and DPC status was not modified by the other significant biomarkers. This observation implies that there was no additive effect among the major biomarkers.

### 3.5. Multimarker Expression Model with Potential Implications of a Second Primary Malignancy

**Figure 4:**
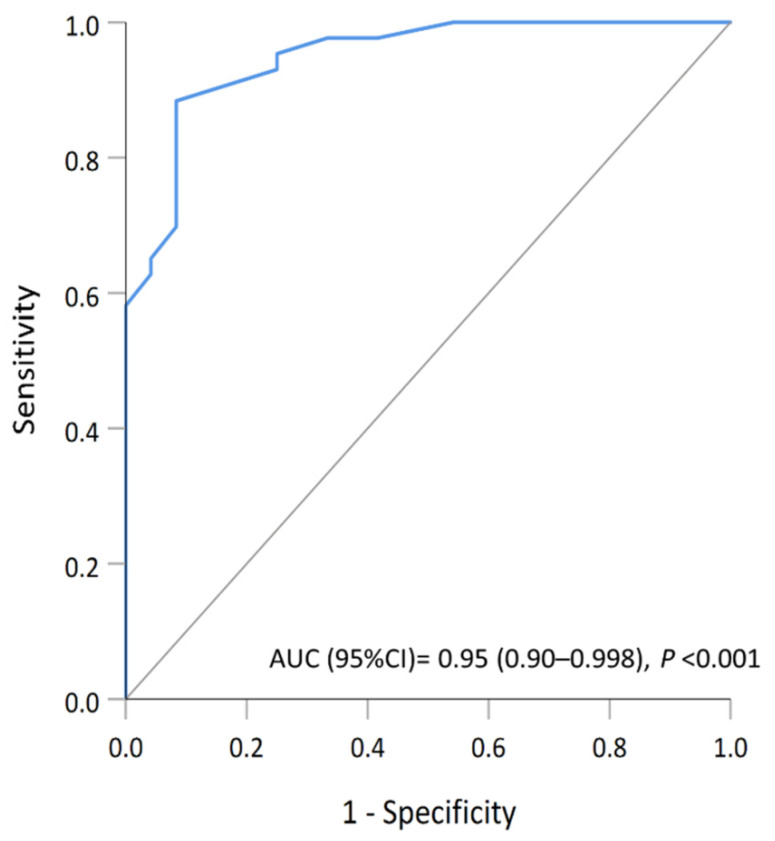
The analysis of the receiving operating characteristics curve demonstrating the ability of the combined predictive model (dMMR phenotype, pRb expression and lack of CDK4 or CDK6) to discriminate the DPC group.

To identify biomarkers that could accurately determine the DPC group, a multivariate analysis of selected variables was carried out. The combined pattern of the dMMR phenotype, the expression of pRb, and the lack of CDK4 or CDK6 was examined to verify the significance in the risk evaluation. To evaluate the performance of the selected multivariate model in distinguishing the status of the DPC, a ROC analysis was performed. The result demonstrated an excellent discrimination performance with the area under the curve of 0.95 (95% CI, 0.90–0.998) (Figure 4). The predictive value for the co-occurrence of nonthyroid malignancy was also assessed. The predicted probability of DPC (ProbDPC) was Equation (1):

ProbDPC = 1/(1 + e^−z^) (1)

where e denotes the exponential function and z denotes the risk score. The risk score of a predictive model of four biomarker signatures was calculated as described in the Equation (2).

z = β0 + β1 × dMMR + β2 × pRb + β3 × CDK4 + β4 × CDK6(2)

with Z equal to risk score, β0 equal to the intercept, and β1 to β4 equal to each biomarker’s coefficient value of each biomarker from multivariate logistic regression analysis [32,33]. In the generation dataset, β0 = −1.087, β1 = 2.336, β2 = 4.138, β3 = −1.671, and β4 = −3.578. Patients with a predicted probability of more than 0.5 are classified as the DPC group.

## 4. Discussion

In previous studies, patients with thyroid cancer have been shown to be associated with a higher risk of nonthyroid malignancies that occur in almost all organ systems [4,5,6,7,9,10,11,12,13,14,15,17,18,19,23,34]. Most of these studies focused on epidemiological trends, while other studies suggested a role for pathophysiological effects in the molecular pathogenesis of second primary malignancy [25,34]. Elucidation of common mechanisms of cancer development has important implications both in diagnostic work and in therapeutic management of thyroid cancer. To our knowledge, this study is the first TMA-based approach to investigate the expression of cell-cycle-related markers and MSI status in patients with thyroid cancer with second primary malignancies.

Detectable biomarkers can be used to guide the optimal frequency of second primary malignancy surveillance. Bunbanjerdsuk et al. reported a set of genes whose expression may serve as prognostic biomarkers for the occurrence of second cancer in head and neck squamous cell carcinomas [35]. Focusing on p16-negative cases, they showed that a multivariate logistic regression model comprising ITPR3, KMT2D, EMILIN1, and patient’s age can accurately predict second cancer occurrence with 88% sensitivity and 75% specificity [35]. In a recent study, thyroid cancer cases with a heterogeneous immunohistochemically stained MMR pattern were likely to be in a state of suboptimal DNA repair capacity, presumed to have caused the co-occurrence of lung cancer [25]. According to previous observations, this technology could be useful in identifying patients with a high risk of a second cancer, based on the analysis of the expression pattern of biomarkers related to the intrinsic characteristics of the thyroid tumor.

### 4.1. Differences in Expression of Cell-Cycle Regulators between Individuals with and without Second Primary Malignancy

Dysregulated cell-cycle progression is one of the hallmarks of malignancy. Previous studies have reported alterations in the expression of cell-cycle regulators in various subtypes of thyroid carcinoma [36,37,38,39,40,41,42,43,44]. Other reports have postulated that cell-cycle regulators could serve as novel predictors of the progression and prognosis of thyroid cancer [38,39,40,41,42,43,45,46].

In the present study, we found associations among single markers and clinicopathological characteristics. Univariate analysis showed that high expression of cyclin D1 and p21 was associated with double primary malignancies (*p* < 0.05). Positivity for pRb and CDK2 (as defined in the Materials and Methods section) was also associated with DPC (*p* < 0.05). On the contrary, the expression of CDK4 and CDK6 was inversely correlated with DPC (*p* < 0.05). A similar expression pattern was observed between PTC and FTC, on the basis of the non-significant interactions. (Table 3)

Protein products of the cyclin D1 and retinoblastoma (Rb) genes play crucial roles in the regulation of the G1/S transition in the cell cycle. Cyclone D1 overexpression has been shown to be associated with aggressive behavior or adverse clinical outcomes in thyroid carcinomas [38,40,41,42,43,45,46]. Overexpression of this positive growth regulator can overwhelm the arrest mechanisms of the normal cell cycle, resulting in uncontrolled cell proliferation. On the other hand, Rb protein immunohistochemistry in malignant thyroid tumors has generated controversial results [31,47,48]. Ferenc et al. reported that overexpression of the Rb protein was found in 83.3% of follicular carcinomas [47]. On the contrary, loss of Rb immunoreactivity in IHC was observed in 82% of malignant thyroid neoplasms in another investigation of Rb expression [31]. The results of our study depict aberrant regulation of several cell-cycle proteins that could be involved in the activation or repression of cell-cycle progression in the development of second malignancies. Thus, these cell-cycle regulators represent potential candidates for new diagnostic and prognostic markers in patients with DPCs.

### 4.2. Possible Clinical Significance of Altered DNA Mismatch Repair Capacity in Patients with Double Primary Malignancies

Microsatellite instability (MSI) is an indicator of decreased fidelity of replication of genomic DNA, and is believed to be associated with genetic defects that promote tumorigenesis. Until now, available data on the evaluation of MMR protein expression in benign and malignant thyroid lesions have been limited [49]. Two previous reports proposed that MSI appears to be an integral part of thyroid carcinogenesis, as evidenced by the downregulated MMR pathway and the correlation with clinical data [50,51]. In addition to prognostic relevance, Mitmaker et al. described a high frequency (63.3%) of MSI in malignant thyroid neoplasms, in agreement with our findings of cases of dMMR (59.7%) [52]. Interestingly, we found a positive correlation between dMMR phenotype and the co-occurrence of nonthyroid malignancy. In our cohort, dMMR was present in thyroid cancer specimens in all kinds of secondary primary malignancies. This finding suggests that MSI status may serve as a potential molecular predictor for high-risk patients.

### 4.3. A Predictive Model Based on the Four Biomarkers (dMMR, pRb, CDK4, and CDK6)

In the current study, the predictive value of immunohistochemical profiles for the co-occurrence of nonthyroid malignancy was also assessed. The significance of the dMMR-like phenotype, the expression of pRb, and the lack of CDK4 or CDK6 was then analyzed, resulting in the development of a four-biomarker signature model. To validate the performance of this model, additional two cases of STC and two cases of DPC were selected using the same criteria as described above. Immunohistochemical analysis of representative proteins was performed. Similar results were found in the four patients, that ProbDPC for two cases of STC were 0.01 and 0.06, respectively; and ProbDPC for two cases of DPC were 0.98 and 0.95, respectively. Among the data set of all 71 patients with thyroid cancer, this model has a sensitivity of 88.9% and a specificity of 69.2% for predicting the occurrence of nonthyroid malignancy. These preliminary results are comparable to the performance of the previous model for head and neck cancer [35].

We identified four tissue-based biomarkers consisting of the dMMR phenotype and three cell-cycle regulators to predict the appearance of DPC. The association between MSI and the cell-cycle regulators cyclin D1 and p21 has been previously described, although the underlying mechanisms remain unclear [53,54]. In particular, the dMMR phenotype did not significantly modify the association between the other significant cell-cycle regulators and the DPC status. However, since these molecular pathways are not mutually exclusive and likely overlap, further analysis is essential to better understand their complex interactions.

This study had several limitations. First, this was a single institution retrospective study; therefore, the small size of the study cohort and the potential selection bias compared to the general population must be taken into account. Furthermore, the importance and robustness of the risk predictive model requires further confirmation with large prospective patient cohorts. Second, the analytical limitations of immunohistochemistry as a method may not have allowed for a precise measurement of protein expression levels. A quantitative molecular study may better integrate the immunohistochemical analyses. Selecting newly eligible cases and demonstrating convincing confirmative results in patients with DPC is necessary. Furthermore, it will be of interest to investigate and confirm the molecular pathway that leads to MMR deficiency and further elucidate the evolution of cancer development.

## 5. Conclusions

A comprehensive molecular analysis of DTC may help to better stratify patients for precision medicine approaches. Our results suggest that dMMR, pRb expression, and lack of CDK4 or CDK6 were significantly associated with the cooccurrence of nonthyroid malignancy. Furthermore, the combined evaluation of a four-biomarker signature model may have the most important predictive implication. The current results serve as a risk assessment for patients with thyroid cancer who develop synchronous or metachronous malignancies. Our study also permits the application of the first tissue-based screening tool for risk stratification, and its further use in active surveillance of thyroid cancer patients. Additionally, therapeutic agents targeting this pathway may play a beneficial role, and clinical studies in this setting are warranted.

## Figures and Tables

**Table 1:** Basic demographics of study cohorts.

Clinical Variable	Single Thyroid Cancer (STC) (*n* = 24) *n* (%)	Double Primary Cancer (DPC) (*n* = 43) *n* (%)
Gender		
Male	3 (12.5)	9 (20.9)
Female	21 (87.5)	34 (79.1)
Age		
<50 years	14 (58.3)	15 (34.9)
≥50 years	10 (41.7)	28 (65.1)
Occurrence interval		
<6 months	NA	9 (20.9)
≥6 months	NA	34 (79.1)
Clinical outcome		
Alive	9 (37.5)	29 (67.4)
Dead	5 (20.8)	8 (18.6)
Not accessible	10 (41.7)	6 (14.0)

NA, not applicable.

**Table 2:** Univariate and multivariate analysis of logistic regression between biomarkers and the status of STC/DPC.

Variable	STC	DPC	Univariate Analysis		Multivariate Analysis	
	(*n* = 24)	(*n* = 43)	Crude OR (95% CI)	*p* Value	Adjusted OR (95% CI)	*p* Value
dMMR	8 (33.3)	32 (74.4)	5.82 (1.95–17.32)	0.002	10.34 (2.17–49.21)	0.003
cyclin D1	13 (54.2)	36 (83.7)	4.35 (1.39–13.61)	0.011		
p21	5 (20.8)	20 (46.5)	3.30 (1.04–10.47)	0.042		
pRb	6 (25.0)	26 (60.5)	4.59 (1.52–13.89)	0.007	62.71 (4.83–814.22)	0.002
p16^INk4a^	3 (12.5)	13 (30.2)	3.03 (0.77–11.98)	0.113		
CDK2	3 (12.5)	19 (44.2)	5.54 (1.43–21.40)	0.013		
CDK4	13 (54.2)	9 (20.9)	0.22 (0.08–0.67)	0.007	0.19 (0.04–0.99)	0.049
CDK6	8 (33.3)	4 (9.3)	0.21 (0.05–0.78)	0.020	0.03 (0.002–0.44)	0.011
E2F1	8 (33.3)	15 (34.9)	1.07 (0.37–3.08)	0.898		
Ki-67	2 (8.3)	14 (32.6)	5.31 (1.09–25.83)	0.039		

STC, single thyroid cancer; DPC, double primary cancer; OR, odds ratio; CI, confidence interval.

**Table 3:** The association between the biomarker and the status of STC/DPC stratified by thyroid tumor subtype.

Variable	Papillary Thyroid Cancer (PTC)			Follicular Thyroid Cancer (FTC)			
	STC (*n* = 13)	DPC (*n* = 35)	OR (95% CI)	STC (*n* = 11)	DPC (*n* = 8)	OR (95% CI)	*p* Interaction
dMMR	4 (30.8)	25 (71.4)	5.62 (1.40–22.53)	4 (36.4)	7 (87.5)	12.25 (1.08–138.99)	0.586
cyclin D1	8 (61.5)	29 (82.9)	3.02 (0.73–12.52)	5 (45.5)	7 (87.5)	8.40 (0.76–93.34)	0.474
p21	2 (15.4)	16 (45.7)	4.63 (0.89–24.04)	3 (27.3)	4 (50.0)	2.67 (0.39–18.17)	0.669
pRb	0 (0.0)	21 (60.0)	NA	6 (54.5)	5 (62.5)	1.39 (0.22–8.92)	0.998
p16^INk4a^	2 (15.4)	13 (37.1)	3.25 (0.62–17.01)	1 (9.1)	0 (0.0)	NA	1.000
CDK2	0 (0.0)	17 (48.6)	NA	3 (27.3)	2 (25.0)	0.89 (0.11–7.11)	0.998
CDK4	4 (30.8)	5 (14.3)	0.38 (0.08–1.70)	9 (81.8)	4 (50.0)	0.22 (0.03–1.75)	0.689
CDK6	0 (0.0)	2 (5.7)	NA	8 (72.7)	2 (25.0)	0.13 (0.02–0.999)	0.999
E2F1	6 (46.2)	12 (34.3)	0.61 (0.17–2.22)	2 (18.2)	3 (37.5)	2.70 (0.33–21.98)	0.236
Ki-67	2 (15.4)	12 (34.3)	2.87 (0.55–15.10)	0 (0.0)	2 (25.0)	NA	0.999

STC, single thyroid cancer; DPC, double primary cancer; OR, odds ratio; CI, confidence interval; NA, not applicable.

**Table 4:** The association between the major biomarkers and the status of STC/DPC stratified by the other major biomarkers.

Variable	dMMR			MMR-Proficient			
	STC (*n* = 8)	DPC (*n* = 32)	OR (95% CI)	STC (*n* = 16)	DPC (*n* = 11)	OR (95% CI)	*p* Interaction
CDK4	3 (37.5)	7 (21.9)	0.47 (0.09–2.45)	10 (62.5)	2 (18.2)	0.13 (0.02–0.84)	0.321
CDK6	2 (25.0)	3 (9.4)	0.31 (0.04–2.28)	6 (37.5)	1 (9.1)	0.17 (0.02–1.65)	0.688
pRb	2 (25.0)	17 (53.1)	3.40 (0.59–19.46)	4 (25.0)	9 (81.8)	13.50 (2.01–90.69)	0.295
**Variable**	**pRb = Positive**			**pRb = Negative**			
	**STC** **(*n* = 6)**	**DPC** **(*n* = 26)**	**OR (95% CI)**	**STC** **(*n* = 18)**	**DPC** **(*n* = 17)**	**OR (95% CI)**	***p* Interaction**
dMMR	2 (33.3)	17 (65.4)	3.78 (0.58–24.75)	6 (33.3)	15 (88.2)	15.00 (2.55–88.17)	0.295
CDK4	6 (100.0)	6 (23.1)	NA	7 (38.9)	3 (17.6)	0.34 (0.07–1.61)	0.998
CDK6	5 (83.3)	4 (15.4)	0.04 (0.00–0.40)	3 (16.7)	0 (0.0)	NA	0.999
**Variable**	**CDK4 = Positive**			**CDK4 = Negative**			
	**STC** **(*n* = 13)**	**DPC** **(*n* = 9)**	**OR (95% CI)**	**STC** **(*n* = 11)**	**DPC** **(*n* = 34)**	**OR (95% CI)**	***p* Interaction**
dMMR	3 (23.1)	7 (77.8)	11.67 (1.53–89.12)	5 (45.5)	25 (73.5)	3.33 (0.81–13.66)	0.321
pRb	6 (46.2)	6 (66.7)	2.33 (0.40–13.61)	0 (0.0)	20 (58.8)	NA	0.998
CDK6	8 (61.5)	1 (11.1)	0.08 (0.01–0.83)	0 (0.0)	3 (8.8)	NA	0.999
**Variable**	**CDK6 = Positive**			**CDK6 = Negative**			
	**STC** **(*n* = 8)**	**DPC** **(*n* = 4)**	**OR (95% CI)**	**STC** **(*n* = 16)**	**DPC** **(*n* = 39)**	**OR (95% CI)**	***p* Interaction**
dMMR	2 (25.0)	3 (75.0)	9.00 (0.56–143.89)	6 (37.5)	29 (74.4)	4.83 (1.40–16.73)	0.688
pRb	5 (62.5)	4 (100.0)	NA	1 (6.3)	22 (56.4)	19.41 (2.33–161.86)	0.999
CDK4	8 (100.0)	1 (25.0)	NA	5 (31.3)	8 (20.5)	0.57 (0.15–2.11)	0.999

STC, single thyroid cancer; DPC, double primary cancer; OR, odds ratio; CI, confidence interval; NA, not applicable.

## Data Availability

Data are available on request due to all institutional restrictions related to patient privacy.

## Supplementary Materials

The following are available online at https://www.mdpi.com/article/10.3390/cancers13215486/s1, Figure S1: Antibody staining control for immunohistochemistry.
