# Supplementary material for: Co-Occurrence of Differentiated Thyroid Cancer and Second Primary Malignancy: Correlation with Expression Profiles of Mismatch Repair Protein and Cell Cycle Regulators
Source: Cancers (Basel). 2021 Oct 31;13(21):5486. doi: 10.3390/cancers13215486 (PMC8582561; doi:10.3390/cancers13215486)
Supplement: Supplementary file 1 [file cancers-13-05486-s001.zip › cancers-1418211-supplementary.pdf]

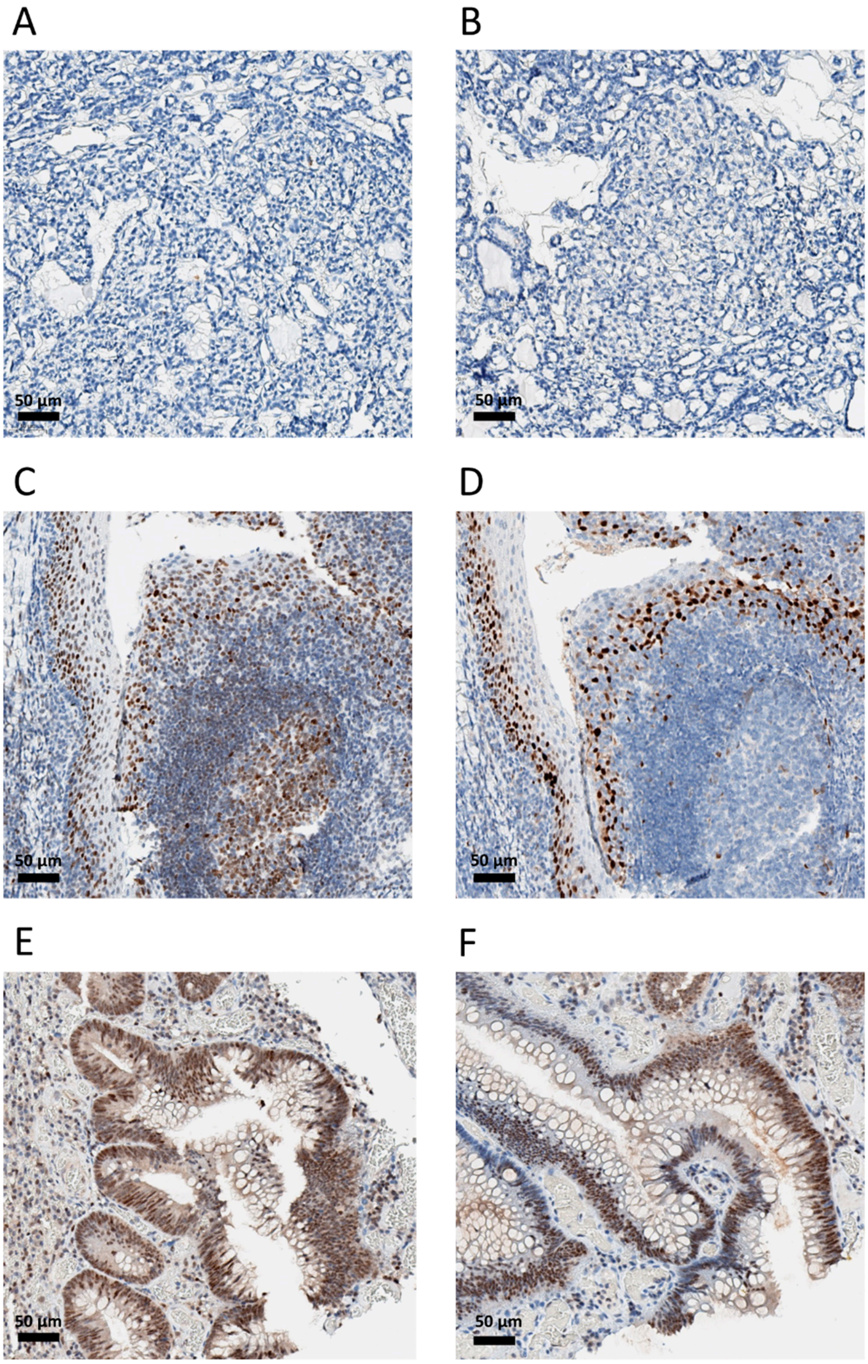

Figure S1. Antibody staining control for immunohistochemistry. Antibody validation was performed with utility of external tissue controls and negative reagent controls. Representative pictures are shown from the separate slides of selected biomarkers: **A** and **B**, Negative controls for CDK4 and CDK6 (follicular carcinoma; no primary antibody used). **C** and **D**, External tissue controls for pRb and cyclin D1 (human tonsil). **E**, Positive tissue control for PMS2 (human colon adenoma). **F**, Positive tissue control for MSH1 (human colon adenoma).
